# Prognostic and Predictive Markers for Early Stage Triple‐Negative Breast Cancer Treated With Platinum‐Based Neoadjuvant Chemotherapy

**DOI:** 10.1002/cam4.70336

**Published:** 2024-10-24

**Authors:** Zhenhui Zhao, Li Li, Mei He, Yan Li, Xiaoping Ma, Bing Zhao

**Affiliations:** ^1^ Breast Internal Medicine Department The 3rd Affiliated Teaching Hospital of XinJiang Medical University (Affiliated Cancer Hospital) Urumqi China

**Keywords:** logistic regression, overall survival (OS), platinum‐based chemotherapy, single nucleotide polymorphisms (SNPs), triple‐negative breast cancer (TNBC)

## Abstract

**Background:**

Emerging evidence has indicated possible efficacy benefit of platinum‐based chemotherapy as neoadjuvant treatment for invasive ductal carcinoma triple‐negative breast cancer (TNBC). However, it has not been endorsed by current guidelines due to highly controversial results.

**Materials and Methods:**

Present study aims to investigate predictive and prognostic roles concerning single nucleotide polymorphisms (SNPs) in *XRCC1* and *BRCA1*, *BRCA2* genes for early stage TNBC patients that received platinum‐based neoadjuvant treatment. We prospectively enrolled women with stage IIB‐IIIB TNBC that had progressed on neoadjuvant taxane and anthracycline‐based chemotherapy at Xinjiang Medical University Affiliated Cancer Hospital. Tumor response and pathological complete response (pCR) rate were assessed. Invasive disease‐free survival (iDFS) and overall survival (OS) were analyzed. Patients' blood samples were subject to Sanger sequencing to genotype *XRCC1* Arg194Trp and Arg399Gln, *BRCA1* s1799949, and *BRCA2* rs206115. Univariate and multivariate logistic regressions were employed to investigate associations between SNPs and clinical characteristics with treatment response and pCR. A total of 45 patients were enrolled.

**Results:**

The cohort showcased ORR of 44.4%, pCR of 28.9%, median iDFS of 22 months, and a 3‐year OS of 73.3%. The A/G and G/G genotypes of *BRCA1* rs1799949, and the T/T genotype of *BRCA2* rs206115 were associated with higher responsive rate. Histologic grade of III and Ki67 expression > 65% were associated with low responsive rate. Moreover, the A/G genotype of *BRCA1* rs1799949 and T/T genotype of *BRCA2* rs206115 correlated to high pCR. The histologic III and T4 stage correlated to inferior iDFS. Carrier of *BRCA1* rs1799949 G/G had the most favorable OS, carriers of A/A showed the poorest OS, and those with A/G genotype showed an intermediate OS.

**Conclusions:**

Platinum‐based chemotherapy might serve as a therapeutic option for TNBC patients who were resistant to anthracycline‐ and taxane‐based neoadjuvant therapy. Our study identified several genetic and clinical features that might function as prognostic and predictive markers.

## 1 Introduction

Triple‐negative breast cancer (TNBC) comprises 10%–20% of all breast cancer (BC) cases, which is the most aggressive BC subtype [1]. Compared with other subtypes, patients with TNBC are characterized by higher rates of visceral and brain metastasizes, more rapid disease progression and higher mortality rate [2]. Approximately half of the patients diagnosed with early‐stage TNBC relapse and 37% die within 5 years after surgery [3]. In the neoadjuvant setting, chemotherapy remains the most widely accepted treatment. Patients achieving a pathological complete response (pCR) revealed longer event‐free survival and overall survival than those not achieving pCR [4]. Despite the strong prognostic significance, only 30%–40% of TNBC patients achieve a pCR upon the standard taxane‐ and anthracycline‐based neoadjuvant chemotherapy [5]. It is clinically crucial to improve the pCR rate by offering more personalized regimen.

It has been reported that 20%–30% of TNBCs harbored *BRCA* gene mutations [6, 7], making them potentially susceptible to compounds that interfere with DNA repair mechanisms such as platinum drugs. Several trials have indicated possible pCR benefit of platinum‐based regimen as neoadjuvant treatment in TNBC patients [8, 9], but increased toxicities were also observed in the platinum‐included arm. Moreover, the role of *BRCA* mutations in predicting efficacy of platinum‐based neoadjuvant treatment remains debated in TNBC [10]. Since the use of platinum‐based chemotherapy demonstrates substantial potential to improve survival of patients with early stage TNBC, finding biomarkers to identify a specific subgroup that can benefit from platinum‐based therapy is an urgent need.

*XRCC1* is a vital gene in the base excision repair (BER) pathway. Two single nucleotide polymorphisms (SNPs) Arg194Trp and Arg399Gln have been reported to alter the structure of the encoding protein, thus affecting the BER process. The two *XRCC1* SNPs have also been associated with cancer susceptibility and sensitivity to platinum‐based chemotherapy [11, 12].

In present study, we interrogated prognostic and predictive role of *XRCC1* Arg194Trp and Arg399Gln as well as two common BRCA variants *BRCA1* rs1799949 and *BRCA2* rs206115 for patients with early stage TNBC who received platinum‐based neoadjuvant treatment after failure to taxane‐ and anthracycline‐based neoadjuvant therapy.

## 2 Materials and Methods

### 2.1 Patients

This study prospectively enrolled patients with BC who received treatment at Xinjiang Medical University Affiliated Cancer Hospital between Jan 2019 and December 2021. The inclusion criteria consist (1) pathologically confirmed invasive ductal carcinoma, If there are enlarged lymph nodes in the area, coarse needle aspiration biopsy is required to determine whether there is tumor metastasis; (2) TNBC confirmed by immunohistochemical (IHC) markers (including ER, PR, Her‐2 and Ki67); (3) Initially diagnosed with stage IIB‐IIIB locally advanced BC. Patients with cT3N0M0 and cT4N0M0 who are resistant to neoadjuvant chemotherapy with anthracycline can also be included in this study. had received 4 cycles of neoadjuvant treatment with taxane‐ and anthracycline‐based chemotherapy, including docetaxel + epirubicin + cyclophosphamide (TEC), docetaxel + cyclophosphamide (TE), and epirubicin + cyclophosphamide plus sequential docetaxel (EC‐T) regimen, with SD or PD as the best response or had achieved PR after 2 cycles of taxane‐ and anthracycline‐based neoadjuvant treatment but showed SD or PD after 4 cycles; (4) With clinical information available, including age, tumor size, lymph node status, histologic grade, with measurable lesion; (5) ECOG score 0–1. Exclusion criteria were (1) inflammatory BC or metastatic BC; (2) The patients had no severe heart, liver or kidney damage before platinum‐based chemotherapy and no contraindications to chemotherapy; (3) distant metastasis.

### 2.2 Specimen Collection

We use EDTA‐containing blood collection tubes to collect 5 mL of peripheral venous blood from patients and store it in a −80°C low‐temperature refrigerator for future use. All patients had peripheral blood samples collected before chemotherapy to avoid the impact of drug treatment on genetic testing results. This research was approved by the Ethics Committee of our hospital, and each subject signed an informed consent form before blood was drawn.

### 2.3 Treatment

When facing patients with anthracycline‐taxane resistance, we will have a discussion among the entire department, and have two doctors with senior professional titles make the decision. If the opinions of the two doctors with senior professional titles conflict, we will select a third doctor with senior professional title to further discuss and decide. Patients received vinorelbine (25 mg/m^2^) intravenously (IV) on Days 1 and 8 every 3 weeks (Q3W) and cisplatin (75 mg/m^2^, divided into 3 days, IV, Q3W). The efficacy of neoadjuvant chemotherapy was evaluated every two cycles. If the condition improved, the next two cycles of neoadjuvant chemotherapy were performed until six cycles were completed before surgery. If the disease progressed, chemotherapy was stopped and radical mastectomy was performed. Further adjuvant treatment measures after surgery were determined based on postoperative pathological results and immunohistochemistry.

### 2.4 Efficacy Assessment

Bilateral breast MRI and bilateral axillary lymph node ultrasound examinations in the bilateral cervical and biclavicular regions were performed every two cycles to determine the efficacy of neoadjuvant chemotherapy with the NP regimen. Tumor response was assessed through RECIST 1.1.

Complete remission (CR): When evaluated as CR, all target lesions and non‐target lesions disappear, and all lymph nodes must be non‐pathological < 10 mm; partial remission (PR): the sum of the diameters of target lesions is reduced by at least 30% compared with the baseline; progressive disease (PD): the sum of the long diameters of target lesions increases by at least 20%, the absolute number increases by 5 mm, and the appearance of new malignant lesions is considered PD; stable disease (SD): the reduction of target lesions does not reach PR, and the increase does not reach PD. CR and PR were considered response to treatment while SD and PD were non‐response. pCR is ypT0N0/ypTisN0MO. When evaluating the efficacy of neoadjuvant chemotherapy, both the primary lesion and lymph nodes are taken into consideration. The absence of invasive cancer in the primary breast lesion and negative regional lymph nodes is defined as pCR. Invasive disease‐free survival (iDFS) was defined as the time from first diagnosis to recurrence of invasive disease. OS was defined as the time from first diagnosis to death due to any reasons. We analyzed the 3‐year iDFS and OS.

### 2.5 Sanger Sequencing and Genotyping

Genome DNA was extracted from patients' peripheral blood samples applying Ezup Column Blood Genomic DNA Purification Kit follwoing manufacturer's protocols (Sangon Biotech, Shanghai, China). Extracted DNA was PCR amplified utilizing specific primers for *XRCC1* Arg194Trp (F: 5′‐ATACTGACCTTGCGGGACCTTAG‐3′, R: 5′‐CCTCTCAACCCTCAGGACACAAG‐3′), Arg399Gln (F:5′‐GCCAACACCCCCA AGTACAG‐3′; R: 5′‐GACCACCTGTGTTCTCCGCTG‐3′), *BRCA1* rs1799949 (F: 5′‐AATCTATCTGCATTAGTAAGGCCTC‐3′; R: 5′‐CAGCAGAAACCTACAACTCAT GG ‐3′), and *BRCA2* rs206115 (F: 5′‐AATCTATCTGCATTAGTAAGGCCTC‐3′; R: 5′‐CAAAACCTTCAAATGGATTCAGTG‐3′). PCR products were subsequently purified employing SanPrep Column DNA Gel Extraction Kit (Sangon Biotech, Shanghai, China) and sequenced on ABI 3730XL DNA sequencer. The targeted SNPs' genotype was then determined.

### 2.6 Statistics Analyses

All statistical analyses were performed utilizing SPSS Statistics 23.0. Clinical characteristics and genotypes were summarized by descriptive statistics. Chi‐squared test was performed to analyze genotype correlations to clinical characteristics. Univariate and multivariate logistic regression analyses were utilized to study association of SNPs with treatment response and pCR. iDFS and OS were estimated utilizing Kaplan–Meier analysis. HR and associated 95% CI were calculated using Cox proportional‐hazards model. Survival differences between subgroups were assessed applying the log‐rank test. Statistical significance was defined by *p* < 0.05.

## 3 Results

### 3.1 Patient Characteristics

A total of 45 women with invasive ductal carcinoma TNBC, who had progressed on taxane‐ and anthracycline‐based neoadjuvant treatment, were included in present study with median age of 45 years (ranging from 30 to 73 years). 51.1% of them presented with a T 3–4 tumor, 88.9% had axillary lymph node metastasis and 33.3% had metastasis in ipsilateral supraclavicular lymph nodes (Table 1). Tumors from 66.7% patients had a histology grade of III. Tumors from 62.2% patients showed Ki67 expression ≥ 30% and 24.4% had the expression ≥ 65%. Thirteen out of 45 patients (28.9%) had received neoadjuvant treatment with EC‐T, 22 (48.9%) had received TEC regimen, and 10 (22.2%) were treated with TE regimen. Among the 45 patients received vinorelbine and cisplatin, 13 and 7 achieved CR and PR respectively. While 18 and 7 patients attained SD and PD, resulting in ORR of 44.4% and pCR rate of 28.9%.

**TABLE 1:** Patient characteristics.

Characteristic	No. of patients
Age	
≤ 35 years	4 (8.9%)
> 35 years	41 (91.1%)
Tumor size	
≤ 2 cm	3 (6.7%)
> 2 cm	42 (93.3%)
T stage	
T1–2	22 (48.9%)
T3‐4	23 (51.1%)
Histology grade	
II	15 (33.3%)
III	30 (66.7%)
Regional LN	
N0–2	30 (66.7%)
N3	15 (33.3%)
Axillary LN	
Negative	5 (11.1%)
Positive	40 (88.9%)
Ki67 expression	
≤ 30%	17 (37.8%)
30%–65%	17 (37.8%)
> 65%	11 (24.4%)
First invasive organ	
Non‐PD	11 (24.4%)
Non‐visceral organ	12 (26.7%)
Visceral organ	22 (48.9%)

### 3.2 Distribution of Different SNP Genotypes

**FIGURE 1:**
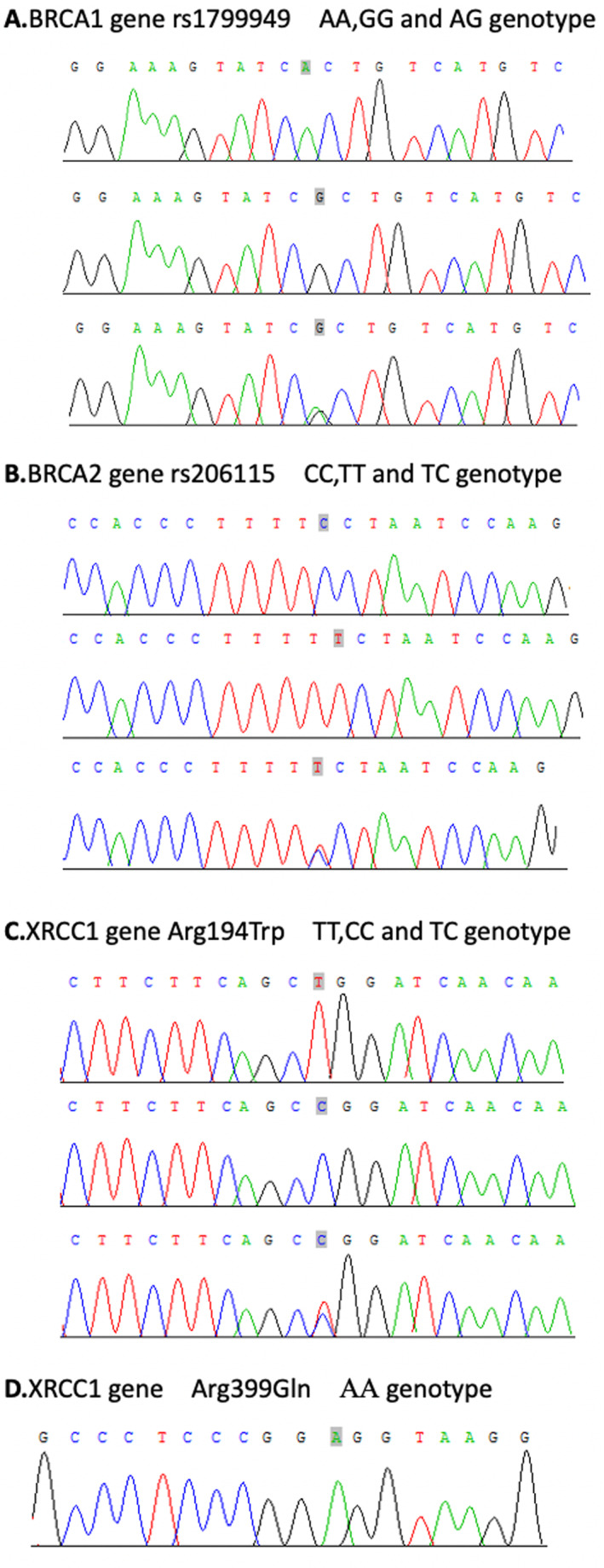
Sequencing results of *BRCA1* gene rs1799949, *BRCA2* gene rs206115, *XRCC1* Arg194Trp and *XRCC1* Arg399Gln.

We Sanger sequenced the *BRCA1* rs1799949, *BRCA2* rs206115, *XRCC1* Arg194Trp, and Arg399Gln in our cohort (Figure 1A–D). Among the 45 patients, 22.2% (10/45), 33.3% (15/45), and 44.5% (20/45) have a genotype of A/A, A/G, and G/G for rs1799949, respectively (Table 2). 33.3% (15/45), 42.2% (19/45), and 24.5% (11/45) patients showed a genotype of T/T, T/C, and C/C for rs206115. The Arg194Trp genotype of C/C, T/C, and T/T accounted for 75.6% (34/45), 17.8% (8/45), and 0.67% (3/45) of the cohort. The genotype of Arg399Gln is A/A in all patients, which was excluded in subsequent analyses.

**TABLE 2:** The association of different genotypes regarding *BRCA1 gene* rs1799949, *BRCA2 gene* rs206115 and *XRCC1 gene* Arg194Trp with patient clinicopathological characteristics.

Characteristic	*BRCA1* rs1799949				*BRCA2* rs206115				*XRCC1* Arg194Trp			
	AA	AG	GG	*p*	CC	TC	TT	*p*	CC	TC	TT	*p*
Age				0.370				0.925				0.882
≤ 35 years	0	1	3		1	2	1		3	1	0	
> 35 years	10	14	17		10	17	14		31	8	2	
Tumor size				0.448				0.921				0.111
≤ 2 cm	1	0	2		1	1	1		1	2	0	
> 2 cm	9	15	18		10	18	14		33	7	2	
T stage				0.146				0.371				**0.026**
T1–2	4	5	13		6	7	9		13	8	1	
T3–4	6	10	7		5	12	6		21	1	1	
Histology grade				0.963				0.798				0.611
II	3	5	7		4	7	4		10	4	1	
III	7	10	13		7	12	11		24	5	1	
Regional LN				0.799				0.362				0.876
N0–2	7	9	14		6	12	12		23	6	1	
N3	3	6	6		5	7	3		11	3	1	
LN												
Negative	1	1	3	0.734	1	3	1	0.682	5	0	0	0.403
Positive	9	14	17		10	16	14		29	9	2	
Ki67 expression												
≤ 30%	3	4	10	0.305	2	7	8	0.439	13	3	1	0.920
> 30% and ≤ 65%	3	6	8		5	7	5		12	4	1	
> 65%	4	5	2		4	5	2		9	2	0	
First invasive organ				0.200				0.499				0.364
Non‐PD	1	4	6		2	4	5		9	1	1	
Non‐visceral organ	1	6	5		5	4	3		7	4	1	
Visceral organ	8	5	9		4	11	7		18	4	0	

*Note:* The highlighted bold values are statistically significant.

### 3.3 The Association of Polymorphisms With Demographic and Clinical Characteristics

We first investigated the association of SNPs with patients' demographic and clinical characteristics. *BRCA1* rs1799949 and *BRCA2* rs206115 were not associated with any characteristics, including age, clinical stage, histologic grade, regional lymph node metastasis, Ki67 expression and first invasive organ (Table 2). While the C/C genotype of *XRCC1* Arg194Trp was significantly associated with a higher proportion of T3‐4 stage than the T/C and TT genotypes (61.8% vs. 11.1% and 50%, *p* = 0.026, Table 2). The XRCC1 Arg194Trp gene polymorphism is associated with tumor stage (*p* < 0.05). Further pairwise comparisons were conducted using the Bonferroni method, and it was found that there was a significant difference in the composition ratio of tumor stages between TT and TC (*p* = 0.007), and that between TC and TT (*p* = 0.200), there was no significant difference between CC and TT (*p* = 0.74).

### 3.4 *BRCA1* , *BRCA2* and *XRCC1* Polymorphisms Associated With Platinum‐Based Neoadjuvant Treatment Efficacy

Next, we assessed the associations of polymorphisms and clinical characteristics with patient's response to platinum‐based neoadjuvant treatment by univariate and multivariate regression analyses (Tables 3 and 4). The multivariate results revealed that carriers of *BRCA1* (rs1799949) A/G (OR [95%CI]: 3.357 [1.161–6.936], *p* = 0.041) and G/G genotype (OR [95%CI]: 5.479 [1.323–9.131], *p* = 0.033) had a higher responsive rate than carriers of A/A genotype. The *BRCA2* (rs206115) genotype of T/T yielded a higher responsive rate (OR [95%CI]: 8.798 [1.577–16.018], *p* = 0.023) than C/C genotype. Moreover, a histologic grade III (OR [95%CI]: 0.186 [0.041–0.833], *p* = 0.028) and Ki67 expression > 65% (OR [95%CI]: 0.113 [0.016–0.821], *p* = 0.031) were also associated with a lower responsive rate.

**TABLE 3:** Association of SNP with response to platinum‐based neoadjuvant chemotherapy.

Genotype	Responder [*n* (%)]	Non‐responder [*n* (%)]	Univariate		Multivariate	
			OR (95% CI)	*p*	OR (95% CI)	*p*
*BRCA1* rs1799949				0.075		0.088
A/A(Ref)	1	9				
A/G	7	8	7.785 (0.788–23.598)	0.079	3.357 (1.161–6.936)	**0.041**
G/G	12	8	13.500 (1.421–28.258)	**0.023**	5.479 (1.323–9.131)	**0.033**
A/G+ G/G	19	16	10.687 (1.220–23.640)	**0.032**		
*BRCA2* rs206115				0.127		0.070
C/C(Ref)	2	9				
T/C	9	10	4.050 (0.685–23.949)	0.123	5.146 (0.938–10.442)	0.055
T/T	9	6	6.750 (1.064–14.838)	**0.043**	8.798 (1.577–16.018)	**0.023**
T/C+T/T	18	16	5.062 (0.950–26.991)	0.058		
*XRCC1* Arg194Trp				0.651		0.342
C/C(Ref)	13	21				
T/C	5	4	2.019 (0.457–8.920)	0.354	7.078 (0.517–26.924)	0.143
T/T	2	0	/	0.999	/	0.999
T/C+T/T	7	4	2.827 (0.690–11.577)	0.149		

*Note:* The highlighted bold values are statistically significant.

**TABLE 4:** Association of clinical characteristics with response to platinum‐based neoadjuvant chemotherapy.

Genotype	Responder [*n* (%)]	Non‐responder [*n* (%)]	Univariate		Multivariate	
			OR (95% CI)	*p*	OR (95% CI)	*p*
Histology grade(III/II)	10/10	20/5	0.250 (0.067–0.931)	**0.039**	0.186 (0.041–0.833)	**0.028**
Ki67 expression				0.131		0.093
≤ 30% (Ref)	10	7				
> 30% and ≤ 65%	8	9	0.622 (0.160–2.416)	0.493	0.383 (0.082–1.788)	0.222
> 65%	2	9	0.156 (0.025–0.952)	**0.044**	0.113 (0.016–0.821)	**0.031**
T stage				0.243		
1 (Ref)	5	5				
2	8	4	2.000 (0.356–11.230)	0.431		
3	3	6	0.500 (0.078–3.210)	0.465		
4	4	10	0.400 (0.073–2.184)	0.290		
N stage				0.284		
0 (Ref)	5	4				
1	8	5	1.280 (0.228–7.187)	0.779		
2	2	6	0.267 (0.034–2.116)	0.211		
3	5	10	0.400 (0.073–2.184)	0.290		
Age (≥ 35/< 35 years)	18/2	23/2	0.783 (0.100–6.108)	0.815		

*Note:* The highlighted bold values are statistically significant.

We evaluated the association of each SNP/genotype and clinical/pathological features with pCR after neoadjuvant platinum‐based chemotherapy (Tables 5 and 6). Multivariate regression analysis showcased that A/G genotype of *BRCA1* (rs1799949) conferred a higher pCR rate versus A/A genotype (OR [95%CI]: 19.582 [1.266‐91.295], *p* = 0.035). Comparing to C/C genotype, T/T of *BRCA2* (rs206115) yielded a higher pCR rate (OR [95% CI]: 27.291 [2.246–105.544], *p* = 0.013). In addition, histologic grade III was associated with lower pCR rate (OR [95% CI]: 0.094 [0.012–0.725], *p* = 0.023).

**TABLE 5:** Correlation between each SNP and pCR of neoadjuvant chemotherapy with platinum‐based drugs.

Genotype	pCR [*n* (%)]	non‐pCR [*n* (%)]	Univariate		Multivariate	
			OR (95%CI)	*p*	OR (95% CI)	*p*
*BRCA1* rs1799949				0.312		0.093
A/A (Ref)	1	9				
A/G	6	9	6.000 (0.596–60.437)	0.128	19.582 (1.266–91.295)	**0.035**
G/G	6	14	3.857 (0.396–37.582)	0.245	5.239 (0.288–55.286)	0.263
A/G+ G/G	12	23	4.696 (0.530–41.568)	0.165		
*BRCA2* rs206115				0.143		**0.035**
C/C (Ref)	1	10				
T/C	5	14	3.571 (0.360–35.454)	0.277	4.719 (0.131–70.234)	0.294
T/T	7	8	8.750 (0.884–86.603)	0.064	27.291 (2.246–105.544)	**0.013**
T/C+T/T	12	22	5.455 (0.621–47.898)	0.126		
*XRCC1* Arg194Trp				0.388		0.320
C/C (Ref)	8	26				
T/C	4	5	2.600 (0.560–12.069)	0.222	6.439 (0.518–79.988)	0.147
T/T	1	1	3.250 (0.182–58.062)	0.423	3.062 (0.030–11.806)	0.635
T/C+T/T	5	6	2.708 (0.650–11.284)	0.171		

*Note:* The highlighted bold values are statistically significant.

**TABLE 6:** Correlation between clinicopathological characteristics and pCR of neoadjuvant chemotherapy with platinum‐based drugs.

Genotype	pCR [*n* (%)]	non‐pCR [*n* (%)]	Univariate		Multivariate	
			OR (95% CI)	*p*	OR (95% CI)	*p*
Histology grade (III/II)	5/8	25/7	0.175 (0.043–0.707)	**0.014**	0.094 (0.012–0.725)	**0.023**
Ki67 expression				0.310		
≤ 30% (Ref)	6	11				
> 30% and ≤ 65%	6	11	0.999 (0.245–4.083)	0.998		
> 65%	1	10	0.183 (0.019–1.799)	0.145		
T stage				0.688		
1 (Ref)	3	7				
2	6	6	2.333 (0.400–13.609)	0.346		
3	0	9	/	0.999		
4	4	10	0.933 (0.157–5.543)	0.939		
N stage				0.362		
0 (Ref)	4	5				
1	5	8	0.781 (0.139–4.387)	0.779		
2	2	6	0.417 (0.053–3.306)	0.407		
3	2	13	0.192 (0.026–1.401)	0.104		
Age (≥ 35/< 35 years)	1/12	3/29	0.968 (0.090–10.381)	0.978		

*Note:* The highlighted bold values are statistically significant.

### 3.5 *BRCA1* , *BRCA2* and *XRCC1* Polymorphisms Associated Survivals Upon Platinum‐Based Neoadjuvant Treatment

**FIGURE 2:**
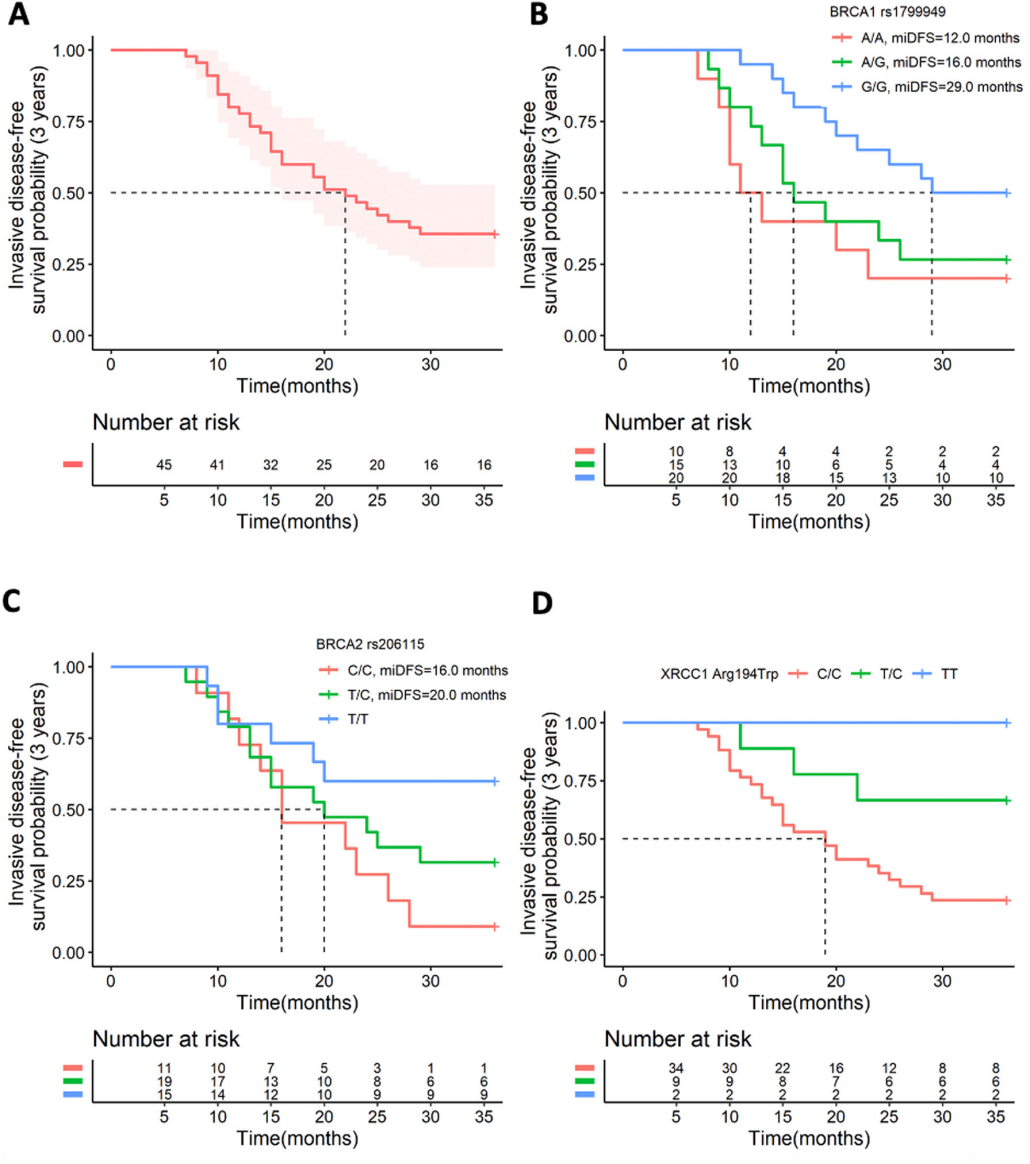
Comparison of invasive disease–free survival (iDFS) among patients with different genotypes. (A) shows the 3‐year iDFS of the population. (B) gives the 3‐year iDFS of different genotypes of *BRCA1* gene rs1799949. (C) provides the 3‐year iDFS of different genotypes of *BRCA2* gene rs206115. (D) shows the 3‐year iDFS of different genotypes of *XRCC1* gene Arg194Trp.

Finally, we interrogated whether these polymorphisms and clinical characteristics were associated with patient's survivals, including OS and iDFS. Median iDFS of the cohort was 22 months (Figure 2A). Median iDFS was 29, 16, and 12 months for carriers of *BRCA1* (rs1799949) G/G, A/G, and A/A, respectively (Figure 2B). Carriers of G/G (HR [95%CI]: 0.138 [0.039–0.489], *p* = 0.002) and A/G (HR [95%CI]: 0.205 [0.058–0.723], *p* = 0.014) elucidated significantly longer iDFS than carriers of A/A, which were revealed by multivariate Cox analysis (Table 5). The median iDFS was not reached for carriers of *BRCA2* (rs206115) T/T, and was 20 and 16 months for carriers of T/C and C/C, respectively (Figure 2C). The T/T genotype yielded significantly longer iDFS than C/C genotype (HR [95%CI]: 0.224 [0.052–0.954], *p* = 0.043) (Table 5). Carriers of T/T for *XRCC1* Arg194Trp did not experience invasive disease until the last follow‐up. Carriers of T/C did not reach median iDFS, and it was 19 months for carriers of C/C (Figure 2D). Differences among genotypes of the polymorphism was not significant (Table 5). Moreover, we identified that histologic grade III (HR [95%CI]: 3.833 [1.327–11.071], *p* = 0.013), T3 (HR [95%CI]: 5.668 [1.179–27.251], *p* = 0.030) and T4 stage (HR [95%CI]: 6.860 [1.505–31.273], *p* = 0.013), and Ki67 expression > 65% (HR [95%CI]: 4.162 [1.181–14.668], *p* = 0.026) were associated with inferior iDFS.

**FIGURE 3:**
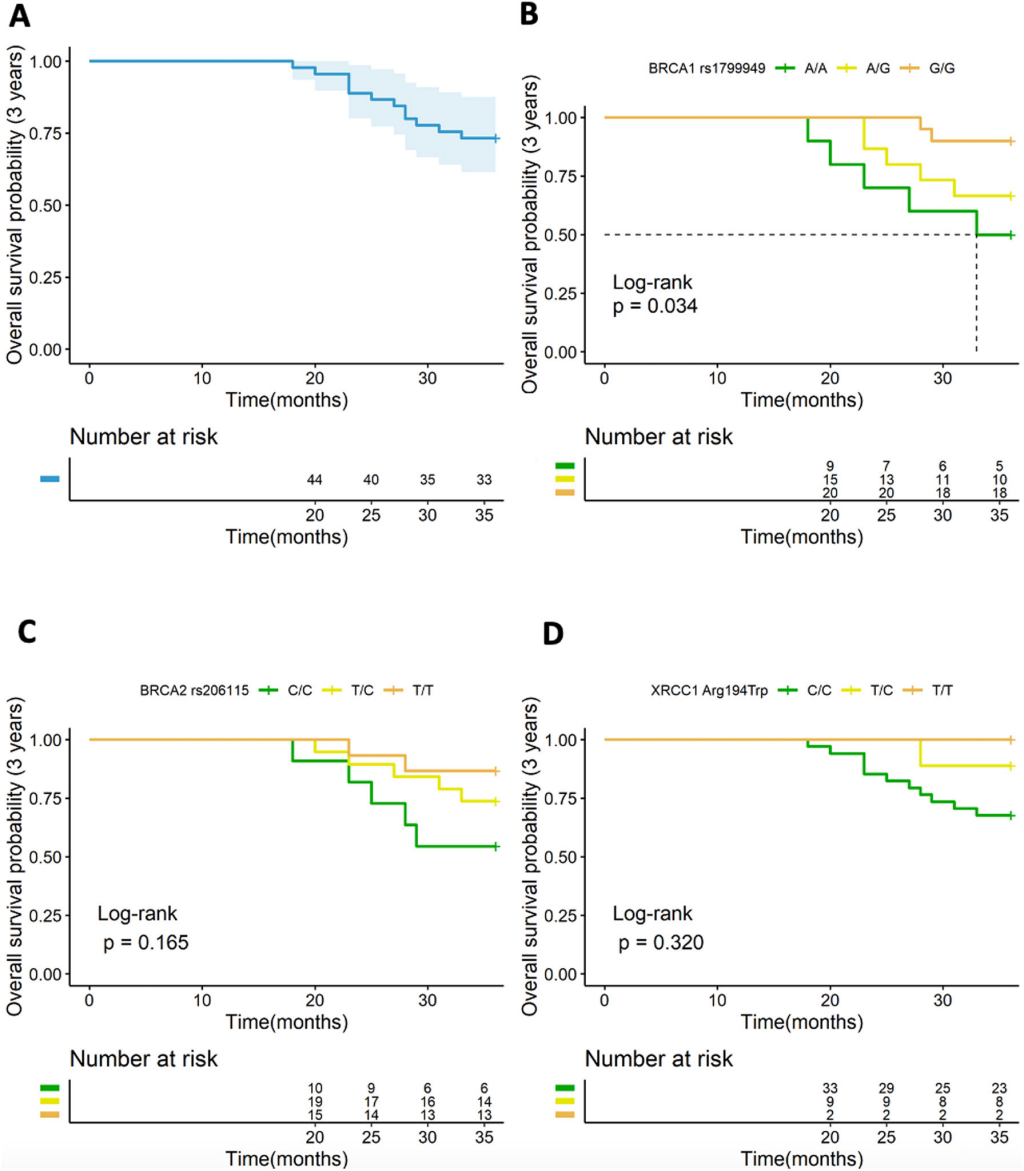
Comparison of overall survival (OS) among patients with different genotypes. (A) shows the 3‐year OS of the population. (B‐D) show the 3‐year OS of different genotypes of *BRCA1* gene rs1799949, *BRCA2* gene rs206115, and *XRCC1* gene Arg194Trp.

The 3‐year OS rate was 73.3% in our cohort (Figure 3A). Median OS was not reached for all 3 genotypes of *BRCA1* rs1799949, *BRCA2* rs206115, and *XRCC1* Arg194Trp (Figure 3B–D). Carrier of *BRCA1* rs1799949 G/G had the most favorable OS, carriers of A/A showcased the poorest OS, and those with A/G genotype gave an intermediate OS (*p* = 0.034) (Figure 3B).

## 4 Discussion

Current study recruited 45 female with early stage TNBC, who had progressed on anthracycline‐ and taxane‐based neoadjuvant treatment. Our data revealed that this subpopulation is identified by large tumor size, high rate of metastasis in axillary lymph nodes, and higher histologic grade as well as Ki67 expression. It advised that TNBC patients with such clinical features have a high risk of developing resistance to taxane‐ and anthracycline‐based neoadjuvant treatment, which represent a neoadjuvant treatment refractory subpopulation. Chemotherapy (CT) containing taxanes and anthracyclines is currently the main systemic treatment option for TNBC. Early emergence of intrinsic or acquired CT resistance is common and is the main obstacle to the successful treatment of TNBC. In the past decade, many mechanisms that may lead to chemotherapy resistance have been discovered, including cancer stem cell (CSC) induction after neoadjuvant chemotherapy (NACT) [13], ATP‐binding cassette (ABC) transporters [14], hypoxia and avoidance of apoptosis [15], tyrosine kinase receptors (EGFR, IGFR1) [16], integrin and metalloproteinase 10 (ADAM10) [17], non‐coding RNA [18], DNA methylation and phosphorylated proteome, including kinase phosphorylation [19] and some pathological molecular pathways such as TGF‐Beta pathway [20], Notch pathway [21], Wnt/β‐catenin pathway [22], Hedgehog (Hh) pathway [23], NF‐kB pathway [24], PTEN and PI3K‐AKT–mTOR pathway [25], JAK/STAT pathway [26], etc.

The standard anthracycline‐ and taxane‐based neoadjuvant chemotherapy yields unsatisfactory pCR rate among TNBC patients. Amounting evidence has illustrated efficacy benefit of platinum‐based regimen. The GeparSixto trial reported a higher pCR rate in patients receiving carboplatin‐based neoadjuvant therapy than those taking neoadjuvant therapy without carboplatin (53.2% vs. 36.9%, *p* = 0.005) [9]. Several meta‐analyses of randomized controlled trials (RCTs) revealed a similar pCR benefit [10, 27]. One recently meta‐analysis including 9 RCTs illustrates that platinum‐based neoadjuvant chemotherapy provided significant improvements in pCR (RR = 1.51, *p* < 0.001), ORR (RR = 1.20, *p* = 0.001), OS (HR = 0.56, *p* < 0.001) and PFS (HR = 0.48, *p* < 0.001) versus non‐platinum neoadjuvant chemotherapy [28]. Current study discovered lower pCR rate of 28.9% than previous reports, which might be due to the fact that patients enrolled in the study had progressed on anthracycline‐ and taxane‐based neoadjuvant chemotherapy. TNBC is sensitive to platinum‐based treatment after neoadjuvant resistance to taxane anthracycline therapy, which is mainly related to the anticancer mechanism of platinum drugs. The most prominent anticancer molecular mechanism of platinum drugs cisplatin and other platinum compounds is to kill cancer cells by damaging DNA, inhibiting DNA synthesis and mitosis, and inducing apoptotic cell death, followed by activation of DNA damage response and induction of mitochondrial apoptosis [29]. The molecular mechanism of platinum drugs in anticancer treatment includes inducing oxidative stress [30], which is characterized by the production of reactive oxygen species and lipid peroxidation. Oxidative stress is one of the most important mechanisms of cisplatin toxicity. Mitochondria are the main targets of cisplatin‐induced oxidative stress, leading to the loss of mitochondrial protein sulfhydryl groups, inhibition of calcium uptake, and reduction of mitochondrial membrane potential [31]. Cisplatin disrupts calcium homeostasis through lipid oxidation and enzyme inhibition, thereby inducing mitochondrial damage, inhibiting mitochondrial function, consuming adenosine triphosphate (ATP) and other cofactors to damage cells, and ultimately leading to cell apoptosis and tissue necrosis [32]. Platinum drugs induce p53 signaling and cell cycle arrest [33], downregulation of oncogenes and anti‐apoptotic proteins, and activation of the intrinsic and extrinsic pathways of apoptosis [34].

Despite the fact that *BRCA* mutation is a predictive biomarker for platinum‐based regimen in advanced TNBC [35, 36], predictive significance of it remains obscure in neoadjuvant setting. The GeparSixto trial shows that additional carboplatin did not increase pCR rate among TNBC patients with germline *BRCA* mutation (65.4% vs. 66.7%), yet yielded a higher pCR (55% vs. 36.4%, OR, 2.14; 95% CI, 1.28–3.58; *p* = 0.004) and disease‐free survival rates (85.3% vs. 73.5%, HR, 0.53; 95% CI, 0.29–0.96; *p* = 0.04) among those without germline *BRCA* mutations [37]. Pooled results from 2 RCTs unravel that the addition of carboplatin in neoadjuvant chemotherapy failed to improve pCR rate in *BRCA*‐mutated TNBC (OR,1.17, 95% CI 0.51–2.67, *p* = 0.711), while the benefit was observed in *BRCA*‐wt TNBC (OR 2.72, 95% CI 1.71–4.32, *p* < 0.001) [10]. However, a post hoc analysis of WSG‐ADAPT TN trial found a higher pCR rate in BRCA‐mutated patients vs. in all others upon neoadjuvant nab‐paclitaxel plus carboplatin (64.3% vs. 34.5%, OR = 3.41, 95% CI: 1.11–10.50; *p* = 0.03). The controversial outcomes suggest some undiscovered factors that impact the efficacy of platinum neoadjuvant chemotherapy in TNBC and merits further exploration [38].

Present investigation identified two common variants *BRCA1* rs1799949 and *BRCA2* rs206115 that are associated with ORR, pCR rate, and iDFS for TNBC patients treated with neoadjuvant platinum‐based chemotherapy. This is the first study associating these two variants with the sensitivity to platinum‐based regimen. Moreover, we also found that histologic grade of III was associated with lower ORR and pCR rates, as well as shorter iDFS. Ki67 expression > 65% was associated with lower ORR and shorter iDFS. A T stage 3/4 was associated with inferior iDFS. The data reveal the potential of integrating these genetic and clinical features to develop a multivariate predictive model, which can be utilized to screen candidates from TNBC patients who may benefit the most from platinum‐based neoadjuvant chemotherapy.

The CT and TT genotypes of *XRCC1* Arg194Trp have been reported to associate with a higher platinum sensitivity in Chinese NSCLC patients [11, 12, 39]. In our study, patients harboring CT/TT had a ORR of 63.6% comparing to that of 38.2% in those with TT genotype. Nevertheless, the difference was not significant (*p* = 0.149). Both of the two patients with TT genotype responded to the neoadjuvant platinum‐based chemotherapy. CT and TT carriers showcased a numerically higher pCR rate than TT carriers (45.5% vs. 23.5%, *p* = 0.171). Lack of statistical significance may due to the small sample size.

Another limit of present study is that the status of pathogenic germline and somatic *BRCA* mutations were not assessed. Moreover, the follow‐up was not long enough to obtain mature OS data. Therefore, further clinical investigations with larger sample sizes and more rigorous design are warranted to better validate prognostic and predictive significance of the markers that found.

## 5 Conclusions

This prospective study shows that platinum‐based chemotherapy may serve as a therapeutic candidate for TNBC patients who developed resistance to anthracycline‐ and taxane‐based neoadjuvant therapy. Present investigation identified several clinical and genetic features that might function as prognostic and predictive biomarkers. Integrating the markers could better stratify patients to select candidates with the optimal efficacy and survivals upon the platinum‐based neoadjuvant therapy.

## Author Contributions

**Zhenhui Zhao:** methodology (equal), resources (equal), validation (lead), visualization (lead), writing – original draft (lead). **Li Li:** data curation (supporting), formal analysis (equal), methodology (equal), validation (equal), writing – original draft (equal). **Mei He:** data curation (equal), methodology (equal), validation (equal), visualization (supporting), writing – review and editing (equal). **Yan Li:** data curation (equal), resources (equal), visualization (equal), writing – original draft (equal). **Xiaoping Ma:** data curation (equal), formal analysis (equal), resources (equal), writing – review and editing (equal). **Bing Zhao:** conceptualization (equal), funding acquisition (equal), resources (equal), supervision (equal), writing – original draft (lead), writing – review and editing (lead).

## Ethics Statement

Current investigation was approved by Ethics committee of the 3rd Affiliated Teaching Hospital of XinJiang Medical University (Affiliated Cancer Hospital) (approval no. K‐2019065).

## Consent

Written informed consent was obtained from all subjects and/or their legal guardian(s).

## Conflicts of Interest

The authors declare no conflicts of interest.

## Data Availability

Data would be available upon appropriate requests to corresponding author.
